# Robotic therapy for the hemiplegic shoulder pain: a pilot study

**DOI:** 10.1186/s12984-020-00674-6

**Published:** 2020-04-22

**Authors:** Ruthber Rodríguez Serrezuela, Mauricio Torres Quezada, Marcia Hernández Zayas, Arquímedes Montoya Pedrón, Daily Milanés Hermosilla, Roberto Sagaró Zamora

**Affiliations:** 1 University Corporation of Hulia, Nevia, Huila Colombia; 2 Universidad de Oriente, Santiago de Cuba, Cuba; 3 Surgical Clinical Hospital “Juan Bruno Zayas”, Santiago de Cuba, Cuba

**Keywords:** Stroke, Neurorehabilitation, Robotic therapy, Hemiplegic shoulder pain

## Abstract

**Backgrounds:**

Exoskeletons development arises with a leading role in neurorehabilitation technologies; however, very few prototypes for upper limbs have been tested, contrasted and duly certified in terms of their effectiveness in clinical environments in order to incorporate into the health system. The purpose of this pilot study was to determine if robotic therapy of Hemiplegic Shoulder Pain (HSP) could lead to functional improvement in terms of diminishing of pain, spasticity, subluxation, the increasing of tone and muscle strength, and the satisfaction degree.

**Methods:**

An experimental study was conducted in 16 patients with painful shoulder post- ischemic stroke in two experimental groups: conventional and robotic therapy. At different stages of its evolution, the robotic therapy effectiveness applied with anti-gravitational movements was evaluated. Clinical trial was developed at the Physical Medicine and Rehabilitation Department of the Surgical Clinical Hospital “Dr. Juan Bruno Zayas Alfonso” in Santiago de Cuba, from September 2016 - March 2018. Among other variables: the presence of humeral scapular subluxation (HSS), pain, spasticity, mobility, tone and muscle strength, and the satisfaction degree were recorded. Results with 95% reliability were compared between admission and third months of treatment. The Mann-Whitney U-Test, Chi-Square and Fisher’s Exact Tests were used as comparison criteria.

**Results:**

Robotic therapy positively influenced in the decrease and annulment of pain and the spasticity degree, reaching a range increase of joint movement and the improvement of muscle tone.

## Introduction

The World Health Organization (WHO) consider neurorehabilitation as an active process where some individuals, with some injury or neurological disease, could achieve the most optimal integral recovery in order to integrate their activities with the environment in the most appropriate approach. Neurorehabilitation technologies have undergone important changes in the last years due to an exponential increase (according to the health systems data) of cerebrovascular accidents (CVA) as the third leading cause of death in the world and high rate of disability.

In Cuba, the disease incidence is estimated at 22,000 cases per year, where about 9725 patients die [1, 2]. It is expected that 70 new strokes occur in the country per day. In 50÷70% of survivors remain sequels, and about a third are unable to fend for themselves, calculating that approximately 75% lose their ability to return to work [3]. This entity represents the fourth national cause of potentially lost life years, with around 11 years for a rate of 86.9 per 1000 inhabitants [4].

Exoskeletons with rehabilitation purposes have reached a great media diffusion and their development occupies a significant number of research groups in the world. However, although more than 100 prototypes have been developed in recent years, only 10 have been clinically tested and they are commercial. In clinical rehabilitation tasks, only 2% are used [5–7]. Nowadays there is not enough clinical evidence, nevertheless some reports include the possibility to improve the functional result after the CVA [8–13].

In the last two decades, some important meta-analyses, including a number of randomized controlled trials, have been performed to investigate the therapeutic effects of upper limb robotic rehabilitation [14–16]. These studies have shown that robotic therapy either produce improvements in the motor control and muscle strength of the paretic arm without side effects or is more effective than conventional therapy in improving upper limb motor function recovery, especially in chronic stroke patients.

The main topics discussed in these investigations are linked to the following aspects: improving motor recovery in distal and proximal joints, the effect of robotic therapy in chronic patients and those design parameters such as power/weight ratio actuators, DOF, accurate, compactness, greater range of motion, safe operation, reliability in all operations, relatively low complexity and low engineering and construction cost, simple fitting and removal, comfort in wearing, and easy maintenance. Some research has deeply studied the combination with other neurorehabilitation technologies (BCI/BMI, FES and tDCs) in recent years.

Although there are well-documented research on the use of exoskeletons in the rehabilitation of the upper limb post-stroke patients, only a few reports have been focused to the treatment of the painful shoulder of the hemiplegic patient using robotic therapy [8, 12, 13].

Hemiplegic Shoulder Pain (HSP) after CVA is a common and debilitating complication. Its incidence varies between 16%, in patients with flaccid paresis and up to 84% in severe paralysis with significant development of spasticity. It may occur in the acute phase (in the first 2 weeks) but more often after two or 3 months, generating greater difficulty in mobilizing the limb, greater disability in transfers, trunk balance and carrying out daily living activities, hindering functional recovery of the upper extremity.

Researchers at Robotics Research Laboratory from the Feinstein Institute for Medical Research [8] conducted an uncontrolled pilot trial in 18 chronic hemiplegic patients using an end-effector exoskeleton. The patients presented glenohumeral subluxation as the only etiology of the painful shoulder, and spasticity was reported in an unspecified number of them. It reported a significant decrease in subluxation, spasticity and an increase in muscle tone as the main results. In their study, however, it is not possible to infer the degree of spasticity of patients during admission and no relationship is established between subluxation and pain, and how the latter changed during treatment. Although an increase in muscle tone is established because of therapeutic treatment, the ranges of active mobility (ROM) are not reported during the beginning and the end of the training.

In a randomized controlled trial with 19 patients (eight who received robotic therapy), after 1÷8 weeks post-stroke, researchers from the University of Guelph [12] analyzed the influence of robotic therapy in the rehabilitation of the upper limb of hemiplegic patients (three patients with flaccid limb) and hemiparetic. No subluxated patients were reported in this study and shoulder pain (light or medium) is attributed to other lesions of the musculoskeletal system. The main results indicate a significant motor recovery in the arm and hand, but no significant changes in pain are reported, and muscle spasticity is not included in the study.

Researchers at Wonkwang University [13] have conducted a study of the effectiveness of an exoskeleton prototype for the rehabilitation of painful shoulder in the hemiplegic patient. The controlled study includes 36 patients divided into two groups, experimental (11 subluxated patients and 14 with spasticity) and control (11 subluxated and 13 spastic patients). The most important results were the decrease in pain between admission and discharge in the robotic therapy group, but not in the control group. The device, however, only incorporates improvements in shoulder abduction, not being successful for internal and external rotations and shoulder flexion/extension. The levels of improvement in patients’ subluxation of the shoulder and the evolution of spasticity during discharge were not reported.

All these results show the inconsistencies of conventional therapies and the possibilities of using robotic exoskeletons. However, these studies have, as a limitation, not to consider the multifactorial genesis of the HSP syndrome. Therefore, the main purpose of our study is to analyze the influence of changes in abnormal muscle tone like flaccid limb, spasticity (mainly in subscapularis and pectoralis muscles) on the evolution of pain as a result of three controlled therapeutic shoulder exercises performed with robotic therapy.

## Methods

An experimental study in patients who survived ischemic stroke with lesion corresponding to the territory irrigated by the middle cerebral artery, who presented HSP syndrome, in order to evaluate the effectiveness of robotic therapy with anti-gravitational movements was performed. The total number of patients with motor deficit who met the selection criteria was included. If the individuals met the inclusion criteria and gave informed consent, they were assessed, then, by an occupational therapist who was blinded to the study and not directly involved with patient care.

Voluntariness, age over 40 years, flaccid and spastic stages (up to grade 2, Modified Ashworth Scale, MAS) of the hemiplegic shoulder and first episode of cerebrovascular disease, body weight and height up to 100 kg and 175 cm respectively, were parameters considered for the inclusion. Some features were excluded: cognitive, sensory, sensitive and communication deficits that made it difficult to understand and follow instructions; pain, motor deficit and/or previous diagnosis of musculoskeletal shoulder disorder, as well as the clinical conditions that contraindicate the rehabilitation treatment. The following were considered as output criteria: voluntary abandonment, appearance of clinical complications that interfered with the study and/or rehabilitation treatment, recurrence of stroke, absence of 3 treatment sessions, and death.

Two stages of evolution were observed in the affected upper extremity: flaccid (flaccidity, tendon reflex, hypotonic, the patient cannot initiate any specific motor function) and spastic (where hypertonia, glenohumeral subluxation, depression, scapula retraction, adduction and internal rotation of the humerus can be found). This spastic phase can be complete, initiating a total synergy, or partial and incomplete. The variables related to the clinical-epidemiological characteristics collected were evolution time, affected hemibody and paralysis degree: hemiparesis/hemiplegia. According to the radiological criteria established by Prevost and Arsenault humeral scapular subluxation (HSS) was considered as Absent, Moderate (< 7 mm) or Severe (> 7 mm) [12].

### Experimental protocol

**Fig. 1:**
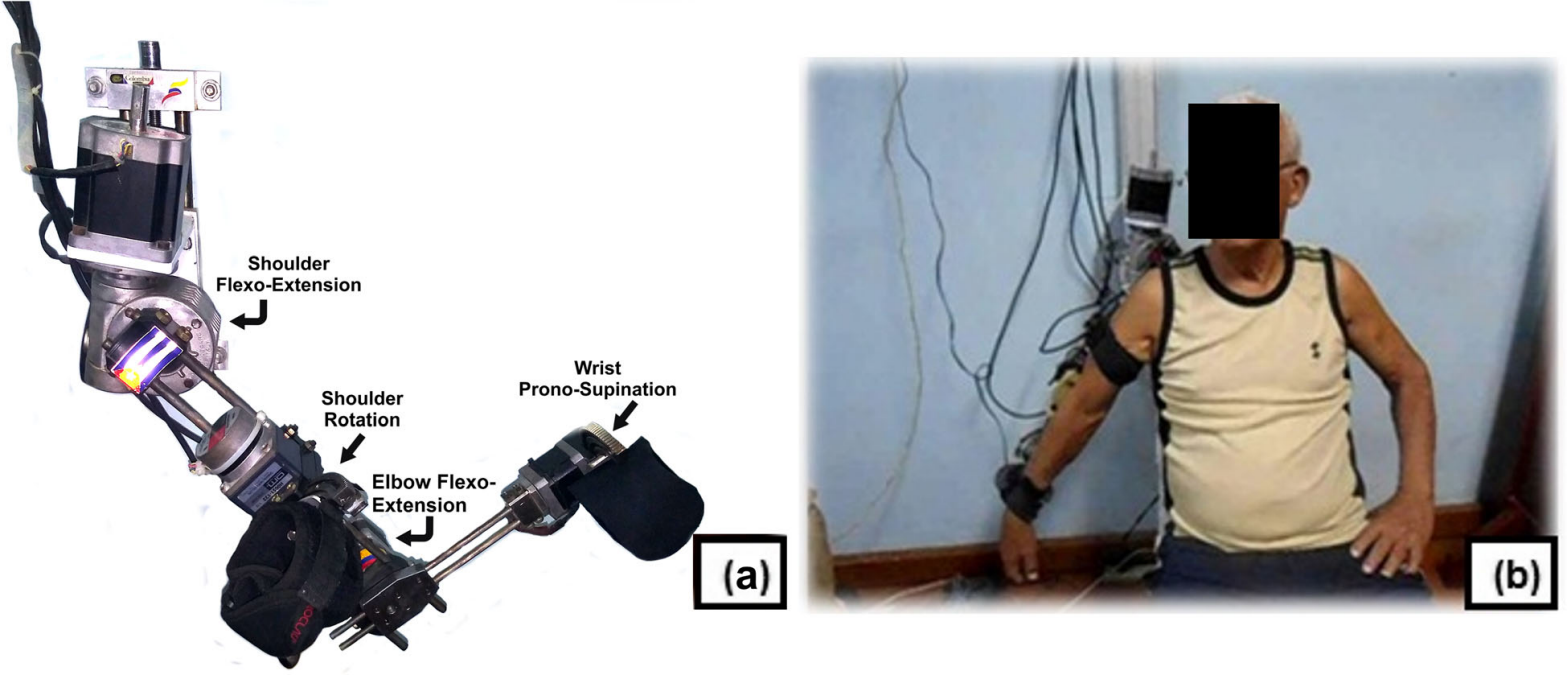
**a** Exoskeleton for upper limb rehabilitation. **b** Patient during abduction training in robotic therapy

Patients with anthropometric characteristics 168 ± 12 cm of height, and 75.7 ± 15.4 kg of weight concluded 3 months of therapy in the grounded robotic platform of 4 freedom degrees developed by the Mechanical and Industrial Engineering Department [17, 18] (Fig. 1). Individuals randomized to the experimental group completed at a rate of 1 effective hour per day, 5 weekly sessions with anti-gravitational movements. The protocol to follow and the experimental procedures, including all the rehabilitation routines and security rules were explained to the participants. No additional visual or audio-visual feedback were supplied.

The therapy was performed in a sitting position with neutral or starting position of the upper extremity. The movement routines selected for the study were: flexion/extension of shoulder and elbow, external and internal rotation and abduction of the upper limb, and prono/supination of wrist. The abduction movement is achieved using shoulder flexion/extension actuator and the counter clock rotation of a patient special-chair design. This particular design (set exoskeleton-chair) allows aligning the rotational axis of the actuator that ensure the flexion/extension movements of the shoulder, with the anatomical axis of the patient for executing abduction/adduction movements. In this way the therapeutic movements of flexion/extension, internal/external rotation and abduction/adduction are performed during the shoulder rehabilitation.

No other kinds of therapy (including thermotherapy, used in those patients of control groups) were programmed for the experimental group.

Exoskeleton is able to permit a physiological range of mobility for selected movements, and the displacement angles were controlled by physiotherapist trough an interface designed for this purpose. Initial values for every patient in each session of the selected routines were fixed considering the maximum pain-tolerable range of motion of the shoulder joint.

**Fig. 2:**
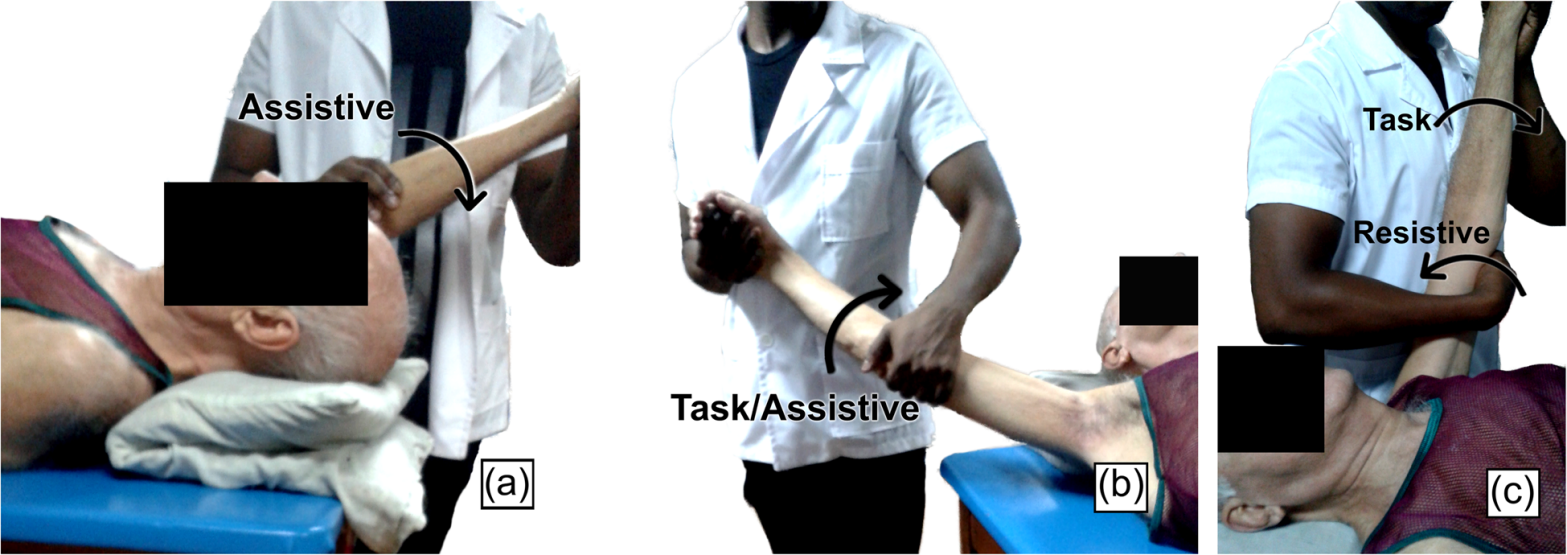
Exercises in conventional therapy. **a** Passive exercise in shoulder flexion assisted by the physiotherapist. **b** Active abduction exercise assisted by the physiotherapist. **c** Active-resistive exercise during the flexion/extension movement of the shoulder

Patients of conventional therapy group (174 ± 6.7 cm of height, 77 ± 13.1 kg of weight) first received superficial thermotherapy [19–22]. The expected therapeutic effects of heat are analgesic, antispasmodic, anti-inflammatory, and the preparation for the exercise. Infrared heat was use for 15 min at a distance of 70÷80 cm according to the affected shoulder and later lymphatic massage and kinesitherapy that included the same movements practiced by robotic therapy group. The kinesitherapy scheme (one effective hour) consisted of the combination of exercises, guided in movement progression and in all its planes. The control group (as well as the robotic therapy group) included hemiplegic and hemiparetic patients, and for this reasons the set of movements involved manual exercises based on Kabat approaches (Fig. 2):

*Assisted Passives* by the physiotherapist, for those patients unable to execute the indicated movement.

*Self-assisted passives* by the patient himself

*Assisted actives* by the physiotherapist, for those patients with certain mobility but unable to complete the movement.

*Resisted (actives)*, for those patients capable of completing the movement, and who, as part of their muscular recovery during the performance of the movements receive a resistive force from the physiotherapist.

These exercises incorporated the nature of the movements selected for the study (i.e. shoulder flexion/extension, internal and external rotation, abduction) and were indicated in correspondence with muscle tone, assessed for all patients according to their classification in the MRC scale and their evolution.

In both experimental and control group, each set of repetitive movement will be divided into 3 time sessions with 10 repetitions each.

### Outcome measures

*Omalgia assessment:* instruments were applied, which allowed weighting the clinical variables indicating their evolution (pain and mobility, muscle tone and strength, active joint movement range, spasticity and satisfaction degree). All of them were recorded at the beginning, every 10 sessions of treatment, with a monthly summary and after 3 months of robotic therapy. Radiological and neurophysiological studies were also performed at the beginning and the end of the treatment.

*Pain assessment:* The severity degree of the painful shoulder was defined [7, 11] in four grades. Severe (0), pain and functional limitation while rest with all movements limited. Moderate (1), pain that intensifies with movement and very light while resting, the movements are painful, but there is no limitation in the joint range. Light (2) without pain at rest, only occurs with rapid movements or under active mobilization and almost normal (3), where only pain appears or limitation to active movements resisted.

*Spasticity Degree:* it was classified according to the well-known numerical Scale of Ashworth Modified (0÷4) [7, 11, 23] that establishes, in ascending order, from normal muscle tone to extreme hypertonia, due to hyperactivity of the mitotic reflex arc.

*Muscular testing:* Based on the application of the Medical Research Council scale (MRC) [16, 24], that establishes different degrees of muscular strength (0÷5) and that in ascending level conceptualizes from the absence of muscular contraction (grade 0) up to normal muscle strength (grade 5).

*Articular assessment of motion range:* Measurement by goniometry was subdivided into three categories, *non-functional shoulder*: active flexion less than 60° and abduction less than 40°; *with improvement*: when actively performing shoulder flexion at 60° and 40° in abduction, and, *functional shoulder*: with ≥90° of flexion and ≥ 75° or more of active abduction [12].

*Degree of subjective general satisfaction:* Likert scale of 10 points ranging from 1 (Nothing) to 10 (A lot) allowing to define the perception of satisfaction level with the improvement degree and its acceptance in relation to the exoskeleton [12].

The results were statistically processed with a 95% reliability when compared between the admission and 3rd month of treatment in order to evaluate its significance level of improvement. The professional package Statgraphics Centurion XV.1 was used and included as comparison criteria, the Mann-Whitney U-Test, Chi-Square and Fisher’s Exact Tests. *P* values of ≤0.05 were considered statistically significant.

### Patients

It was included as a universe for the total number of patients with motor deficit (hemiplegia/hemiparesis) of ischemic cause with lesion corresponding to the territory of the middle cerebral artery,, which presented painful shoulder in flaccid or spastic stages and which complied with the selection criteria. In a group of 18 patients evaluated, two patients did not meet the inclusion criteria.

The 16 patients selected for the study were randomly divided into two groups: one of robotic therapy or experimental group, and one of conventional therapy or control group. A physiotherapist unrelated to the study randomized the participants into one of two groups using a random number Table. A research technician from Universidad de Oriente collected the biomechanical and progress data from the robotic system at admission and discharge. An occupational therapist blinded to patient allocation administered the outcome measures at admission and discharge.

The clinical protocol was applied to eight patients, all with medical diagnosis of HSP, in the pilot study five men and three women, with an average age of 63.4 ± 15.9 years were included. The control group consisted of 4 men and 4 women, with an average age of 64 ± 19.7. Tables 1 and 2 show the general characteristics of the patients included in both study groups.

**Table 1:** General characteristics of the patients involved in the study. Robotic therapy group

Patient	Gender	Age	Evolution from CVA (months)	Affected hemisphere
S1	M	57	7	left
S2	M	71	1	right
S3	F	57	4	left
S4	M	64	2	left
S5	F	64	14	left
S6	M	68	14	right
S7	M	66	4	left
S8	F	76	4	left

**Table 2:** General characteristics of the patients involved in the study. Control group

Patient	Gender	Age	Evolution from CVA (months)	Affected hemisphere
C1	F	77	3	right
C2	F	67	4	left
C3	F	35	4	right
C4	M	65	6	right
C5	F	68	3	left
C6	M	71	6	right
C7	M	62	4	left
C8	M	69	4	left

Tables 3 and 4 show the evaluation indices during admission for each patient in both groups according to the declared measurement instruments. As it can be observed, both groups of patients respond heterogeneously to the different etiologies of the painful shoulder, spasticity degree, muscle tone and severity of the painful shoulder.

**Table 3:** Evaluative indices of patients in the conventional therapy group during admission

Patient	ESS^1^ (mm)	MAS^2^	MRC^3^	Painful shoulder severity ^4^	AJM^5^
C1	–	2	0	2	Not Functional
C2	–	2	1	1	Not functional
C3	20 ± 0,4	2	2	0	Not functional
C4	–	3	2	0	Not functional
C5	–	1	1	1	Not functional
C6	–	2	0	1	Not Functional
C7	–	1	1	1	Not functional
C8	–	2	1	1	Not functional

Where:

^1^ Evaluation of shoulder stability as measured in mm. (single examiner, blinded to design)

^2^ Modified Ashworth Scale

^3^ Muscular evaluation according to the scale of the Medical Research Council

^4^ Degree of Painful shoulder severity

^5^ Degree of functionality of active joint movement according to [12]

**Table 4:** Evaluative indices of patients in the robotic therapy group during admission

Patient	ESS^1^ (mm)	MAS^2^	MRC^3^	Painful shoulder severity^4^	AJM^5^
S1	–	1	2	2	Functional
S2	–	Flaccid	0	1	Not Functional
S3	–	0	1	0	Not Functional
S4	–	2	1	1	Not Functional
S5	25 ± 0,5	1	2	2	Not Functional
S6	–	1	2	2	Not Functional
S7	–	2	3	1	Not Functional
S8	–	0	2	1	Not Functional

Where:

^1^ Evaluation of shoulder stability as measured in mm. (single examiner, blinded to design)

^2^ Modified Ashworth Scale

^3^ Muscular evaluation according to the scale of the Medical Research Council

^4^ Degree of Painful shoulder severity

^5^ Degree of functionality of active joint movement according to [12]

In the experimental group, as a painful shoulder etiology, one of the patients presented severe humeral scapular subluxation (HSS, (measured by a single examiner who was not aware of the point of the study)) while in the rest, the existence of the painful shoulder without subluxation was confirmed (SHSS, neurological etiology or other osteomioarticular causes). On the other hand, in the conventional therapy group, one of the patients showed severe scapula-humeral subluxation while in the rest the existence of the painful shoulder without subluxation was confirmed. Thus in the 88% of patients involved in the study, HSS was absent (by clinical and radiological test), it was confirmed that this etiology was not the main cause of HSP in both groups concerning to this study.

## Results

Tables 5 and 6 show comparatively the main indicators obtained as a result of the application of both therapies in the pilot study.

**Table 5:** Evaluative indices after 3 months of conventional therapy

Patient	ESS^1^ (mm)	MAS^2^	MRC^3^	Painful shoulder severity ^4^	AJM^5^	Satisfaction Degree^6^
C1	–	1	2	No (2 months)	Not functional	5
C2	–	2	2	No (3 months)	Not functional	6
C3	20 ± 0,4	1	3	No (2 months)	Functional	6
C4	–	3	2	1	Not functional	5
C5	–	1	2	No (2 months)	Not functional	7
C6	–	2	2	No (3 months)	Not functional	5
C7	–	1	3	No (2 months)	Not functional	6
C8	–	2	2	No (2 months)	Not functional	5

Where:

^1^ Evaluation of shoulder stability as measured in mm. (single examiner, blinded to design)

^2^ Modified Ashworth Scale

^3^ Muscular evaluation according to the scale of the Medical Research Council

^4^ Degree of Painful shoulder severity

^5^ Degree of functionality of active joint movement according to [12]

^6^ Satisfaction degree of Likert scale

**Table 6:** Evaluative indices after 3 months of robotic therapy

Patient	ESS^1^ (mm)	MAS^2^	MRC^3^	Painful shoulder severity^4^	AJM^5^	Satisfaction Degree^6^
S1	–	0	4^+^	No (20 sessions)	Functional	10
S2	–	0	4^+^	No (20 sessions)	Functional	10
S3	–	0	4^+^	No (20 sessions)	Functional	10
S4	–	1	3	No (1 month)	Functional	9
S5	15 ± 0,3	1	2	No (20 sessions)	Functional	9
S6	–	1	4	No (20 sessions)	Functional	10
S7	–	1	4	No (1 month)	Functional	9
S8	–	0	4	No (20 sessions)	Functional	10

Where:

^1^ Evaluation of shoulder stability as measured in mm. (single examiner, blinded to design)

^2^ Modified Ashworth Scale

^3^ Muscular evaluation according to the scale of the Medical Research Council

^4^ Degree of Painful shoulder severity

^5^ Degree of functionality of active joint movement according to [12]

^6^ Satisfaction degree of Likert scale

It can be seen after 3 months of robotic therapy, patients experienced a significant recovery in relation to the control group.

The results obtained from the different indicators, it is deductible that the patients who received robotic rehabilitation exhibited higher rates of motor recovery. From Tables 5 and 6 it is possible to establish the comparison between both groups, in such terms. It should be noted that in relation to the treatment of spasticity, robotic therapy was more consistent in its reduction, with a great influence in the range of active joint mobility and muscle tone increase.

**Fig. 3:**
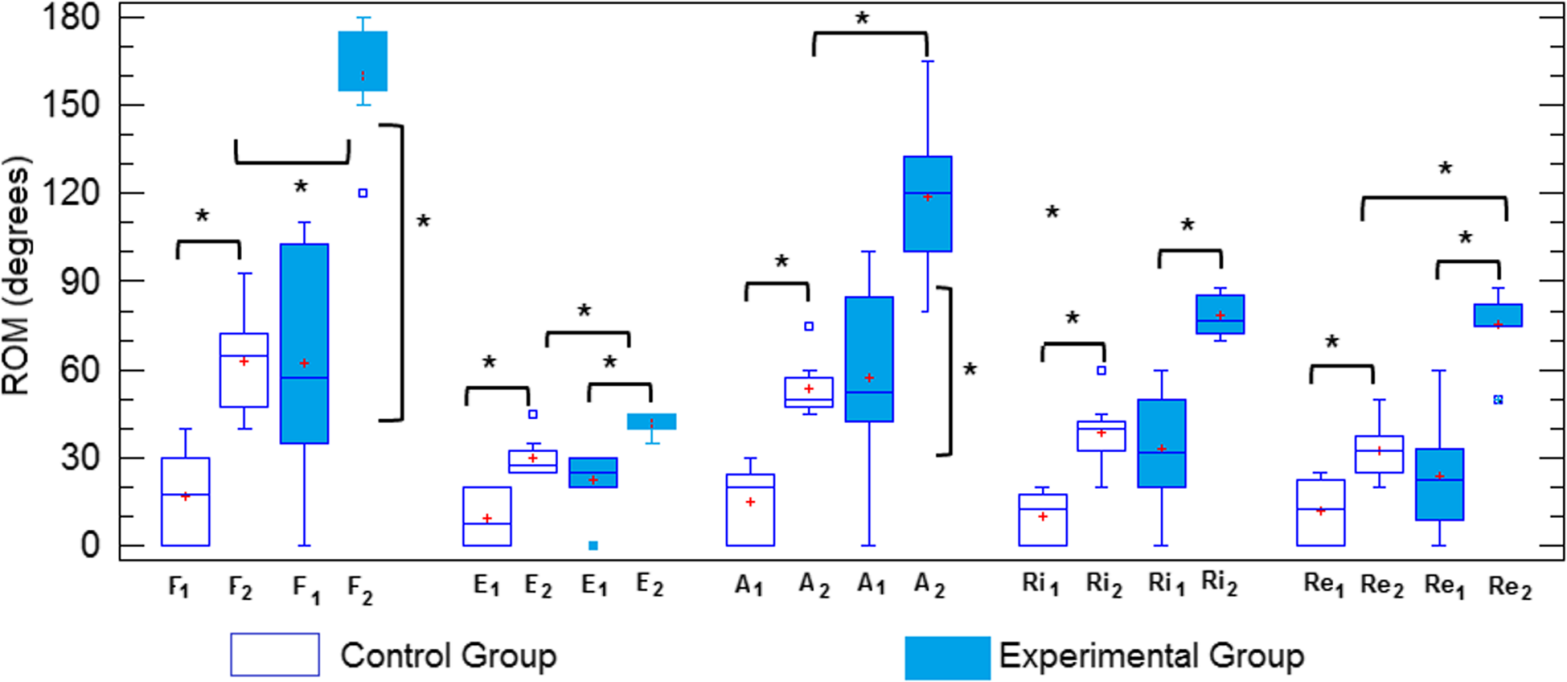
Range of joint movement (ROM) of patients in both therapy group (1, admission, 2, discharge, F-flexion, E-extension, A-Abduction, Ri-Internal rotation, Re- External Rotation- * *p* < 0.05)

An important result is that patients in this group experienced a pain decrease until its disappearance in just 20 sessions and that undoubtedly favors a better balance of all rehabilitation indicators. Other important improvements with the use of robotic therapy were obtained in muscular tone and the increase of ROM. Figure 3 (box plot) and Table 7 show comparatively the range of joint mobility for both groups during admission and after 3 months of treatment for all therapeutical movements.

**Table 7:** Changes in outcome measures (ROM) observed in experimental and control groups after the intervention

	Robotic Therapy (*n* = 8)			Control Group (n = 8)			Mean Change*	Between groups
	Admission	Discharge	*p*-value	Admission	Discharge	p-value	ROM	p-value
Flexion	62.5 ± 39.5	160 ± 19	0.00002	16.8 ± 16	62.7 ± 17	0.001	97.1	0.0008
Extension	23.2 ± 11	41.6 ± 3.5	0.0004	10.1 ± 9.3	30 ± 7	0.007	11.6	0.008
Abduction	57.5 ± 33	118.7 ± 26	0.004	14.75 ± 12	53.7 ± 10	0.0006	65	0.0008
Rot.int	37.6 ± 23	78.3 ± 7	0.0002	10 ± 8	38.5 ± 11.5	0.0006	39.6	0.0006
Rot. ext.	26 ± 20	75.2 ± 11	0.00002	9.7 ± 9.3	31.8 ± 10	0.0043	42.7	0,001

Rot.int. - Internal rotation.

Rot.ext. –External rotation.

(*) Mean difference between discharges of both groups.

## Discussion

It can be seen in Table 1 that the patients in experimental group have an average of 6 months after the stroke (three of them are chronic patients, who entered late to the robotic therapy) and 4 months for the conventional therapy group (Table 2). Considering the results of robotic rehabilitation, it can be said that its application is quite promising in patients with a few weeks of post-stroke evolution and immobilization of the affected limb. However, it is possible to obtain satisfactory results in chronic patients, whose prognosis for rehabilitation are unfavorable,, as mentioned above, it is one of the most discussed topics related to the application of robotic therapy.

The robotic therapy showed in this study is very effective in the pain treatment if it is compared with the results obtained with the conventional therapy. The authors consider this result linked with the efficacy in the treatment of spasticity and musculoskeletal alterations as the most involved elements in the genesis of HSP [24–29].

In all patients of robotic therapy group, the pain disappeared between 22.5 ± 4.5 treatment sessions, result that remained during the following evaluations, while for patients with conventional therapy the pain ceases after 63.7 ± 19.7 sessions. One patients of this last group remained at discharge with persisted pain [*p* < 0.0012 between both groups]. This result has a great importance in this study since the HSP reduces participation in functional activities and in the rehabilitation process. For conventional treatments, after hospital discharge it predicts a poor functional recovery of the arm, a longer duration of admission and the percentage of patients who are discharged to their home is lower [24, 30–32].

**Fig. 4:**
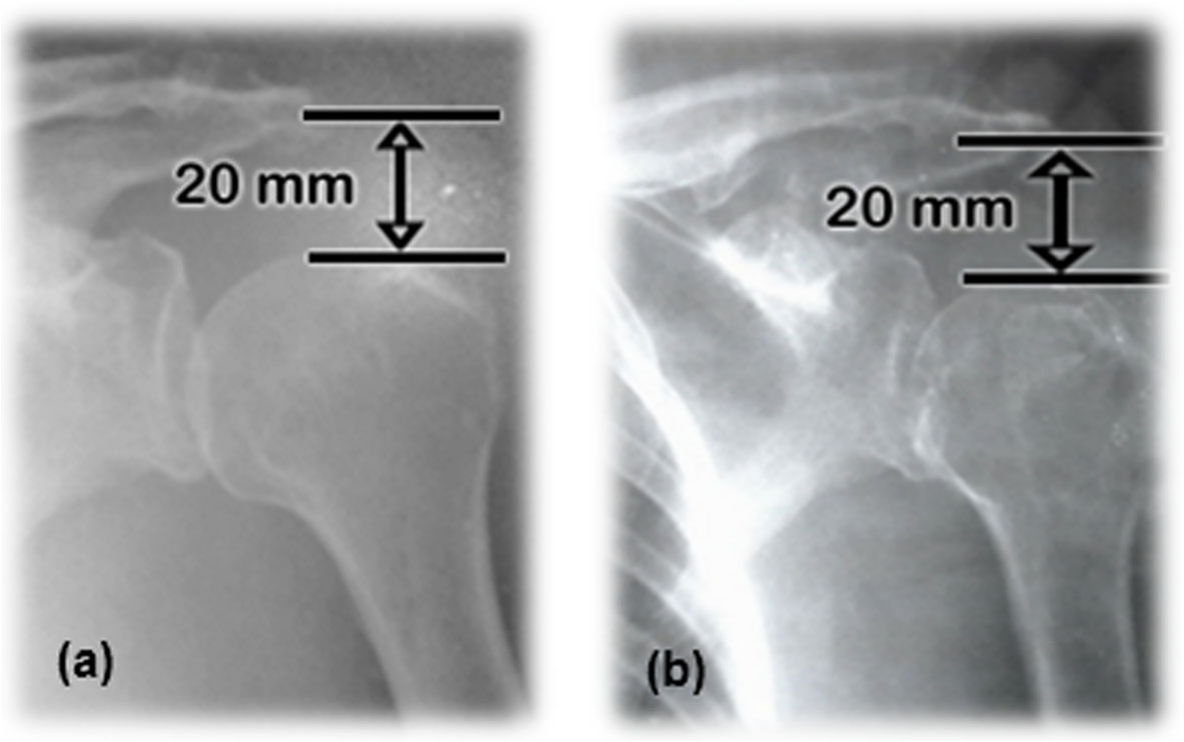
HSS. (**a**) The patient C3 from control group during admission. (**b**) The patient C3 from control group after 3 months of conventional therapy

Although there were few analyzed cases, another interesting result, was related to subluxation as one of the etiologies of the painful shoulder of the hemiplegic patient. Figure 4 shows comparatively the subluxation degree of the patient treated with conventional therapy exercises after 3 months. It should be noted that conventional therapy did not cause favorable changes in the subluxation magnitude.

**Fig. 5:**
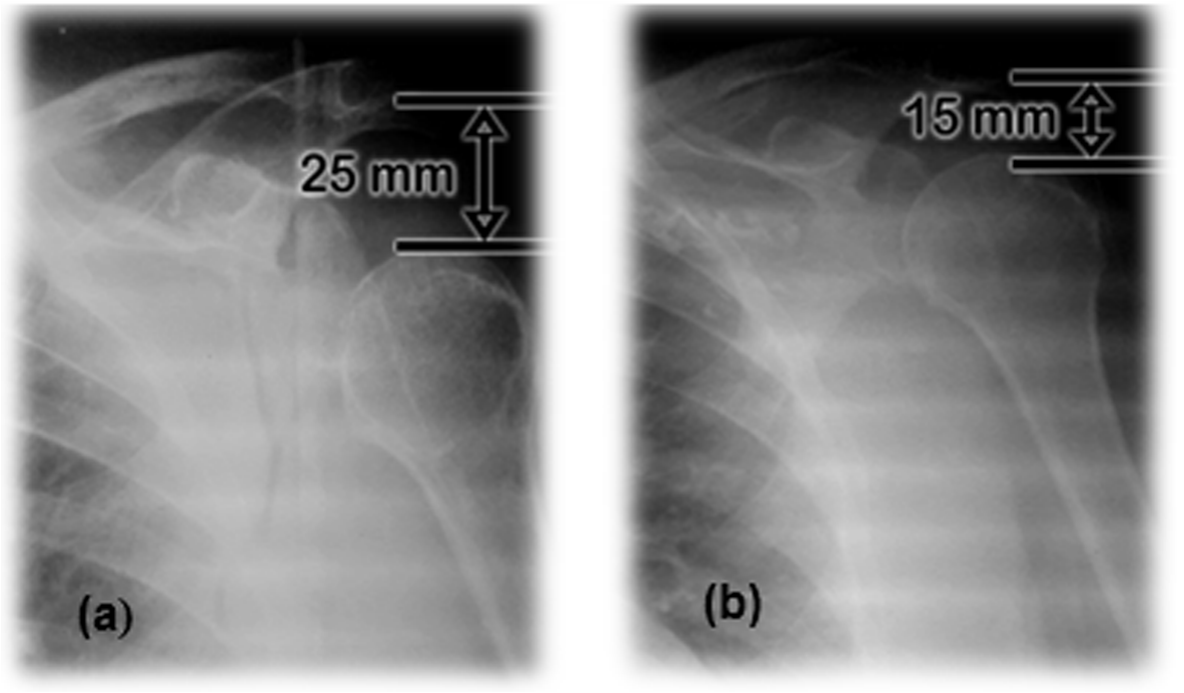
HSS. **a** The patient from robotic therapy group during admission. **b** The patient from experimental group after 3 months of robotic therapy

On the other hand, robotic therapy caused a decrease in the magnitude of subluxated shoulder of 10 mm after 3 months (Fig. 5).

This patient (S5), arrived to admission in a hemiparetic condition, with a time of evolution of 14 months after the stroke, spasticity degree MAS = 1and a large scapula-humeral subluxation. This is a clear example of how much the HSP delays the rehabilitation of the patient, given that 14 months before entering the study group she showed a very discreet rehabilitation degree. The results obtained in this chronic patient denoted the advantages of robotic rehabilitation even in these cases.

Regarding the treatment of spasticity, the results of the conventional treatment were discrete, which meant that there was an improvement in two of the patients, precisely these two patients, showing an enhancement in the treatment of their spasticity, (moderate to mild hypertonia), they presented successively a recovery of muscle tone and range of mobility. The rest kept their level of spasticity, and one of them worsened (*p* < 0.45).

The systematic anti-gravity training in the exoskeleton caused, however, a reduction and control of spasticity in all cases (four as normal and four with light level, *p* < 0.02). Between both groups MAS scores improved significantly (*p* < 0.003).

This result suggests the importance of controlling and reducing spasticity as the main cause that develops the pattern of pain in the shoulder. Precisely muscle spasticity has been associated with immobility to post CVA, and this study suggests how to treat it with a combination of precise and controlled movements that gradually gain intensity. In this way, it is possible to control the pain and achieve a greater motor recovery evidenced with an increase in muscle tone and ROM.

Furthermore, it is significant, the motor rehabilitation in the flaccid patient (whose prognosis is always unfavorable and generally continues until develops spasticity). This patient who experienced an important recovery did not develop spasticity. The same happened with those patients with normal muscle tone during admission.

Other important results are related with the improvement of the range of articular movement (ROM) and muscular tone.

Ranges of joint mobility below those functional, spasticity and absence of muscle tone characterized both groups during admission. As it is known, in the stroke population involved in this kind of experimental studies, there are differences between in general characteristics according to spasticity degree, muscular strength and joint mobility. In this heterogeneity groups exoskeletons can be more helpful and appropriate treatment option for a customized therapy.

In this sense, from Table 7 it can be concluded that the robotic therapy group presented a greater heterogeneity than the control group in ranges of active movement. For example patient S1 (with functional shoulder), who also presented during admission mild pain and hypertonia, MRC = 2 and a favorable prognosis of rehabilitation, and patients S6 y S7 with functional values in flexion shoulder. However the rest of the patients of this experimental group presented high degrees of functional disability.

The increase in joint mobility experienced by patients in the robotic therapy group significantly exceeded those achieved by patients in the control group, in which only one of the patients meets the functionality criteria established in this work. The continuous and significant gradual increase in active joint movement (ROM) of these patients from the admission is the result largely of the control and reduction of spasticity that limits, as it is known the active flexion, abduction and external rotation patterns and the consequent early elimination of pain. These aspects, which however have little or no change during conventional therapy delayed pain elimination, that take place in a period of time that undermines the prediction for better rehabilitation rates [33, 34–36].

From Fig. 3, it is deductible that both rehabilitative treatments were effective. From Table 7 it can be observed that ROM improvements were statistically significant inside both groups, as well as the difference between them, for each of the therapeutic movements involved. However, it should be noted that despite its better ROM indicators during admission, in the functional recovery of the shoulder, the robotic therapy group showed greater increases in the range of joint mobility at the end of the treatment.

Table 7 shows the mean difference during the discharge between both groups for the different movements involved. As noted, such a difference between both treatments is very significant. Despite heterogeneity in the experimental group, robotic therapy achieved an average increase of 97 degrees in shoulder flexion, 65 degrees in abduction and about 40 degrees in rotations, far away from the improvements provided by the conventional therapy.

In this regard, it is worth highlighting those cases of the experimental group whose indicators during admission were comparable to those of the conventional group and nonetheless reach recovery rates on par with the rest of the experimental group, for example the patient with a flaccid limb and the chronic patient S5, who receiving prior conventional therapy entered the experimental group with severe subluxation, spasticity, even with pain and very discrete degrees of joint mobility.

This effect could be explained in how the decrease in spasticity and pain allow a gradual increase in the arcs of articular mobility of the shoulder in patients from the experimental group.

Another important outcome is referred to the recovery of motor capacity expressed in the evaluation of muscle strength from admission and during treatment. From Tables 3 and 5 it is deduced that conventional therapy after 3 months of treatment, although it experienced changes with statistically significance (*p* < 0.002) in muscle tone patients did not show an important recovery as robotic therapy (Tables 4 and 6). There was statistically difference between groups (*p* < 0.0014).

It can be concluded that robotic therapy in general prompted an increase in muscle strength in the patients (according to the reported results in the MRC scale). Only patient S5, who showed since admission to 3 months a value of muscular strength MRC = 2 was the exception, coincidentally being the patient with an evolution time of 14 months and a great subluxation in the left arm, aspects that seem to have influenced in the recovery of the muscle tone.

In general, a greater muscular strength can be observed associated with those patients who achieve greater active ranges of joint movement with robotic therapy, as it has been suggested by other authors [10, 32, 34, 35, 37].

Considering the nature of such pathologies, it is noteworthy the motivation and enthusiasm of the patients during the treatment with robotic therapy. A great satisfaction degree of the people being treated (Table 6), constitutes an indicator not only from the patient’s satisfaction with the result of the therapy, but also constitutes an aspect that positively influences the process of neurorrehabilitación of the hemiplegic patient with the use of exoskeletons.

Finally, all the functional and motor achievements reached by the patients from experimental group were sustained over time, showing in this way, the potential of such treatment in the early recovery of the hemiplegic shoulder.

Although it was not the object of this study, significant results were reported in the rehabilitation of the different indicators in the elbow joint and to a lesser extent of the wrist [38], in addition to very positive impacts on the concentration and the physical capacity of the patients.

## Conclusions

The outcomes of this study favored robotic therapy over conventional therapy in an early elimination of pain in a significant way (20÷30 sessions), which enables the obtaining of better rehabilitation indicators. It means that it is possible to control pain through reducing shoulder spasticity using a proper upper limb exoskeleton therapy.

As it has been reported in the scarce literature on shoulder subluxation in the hemiplegic patient, a rapid and significant decrease in the magnitude of subluxation even in chronic patients (evolution times greater than 6 months) was obtained. In this sense, it is worth mentioning the attainment rehabilitation indices in patients with different evolution times for different etiologies of the painful shoulder of the hemiplegic patient.

Our findings and outcome results were adjusted for the data for this study, even when small sample size was used. A large number of patients would allow more conclusive results than those presented here.

These findings suggest that the lack of mobility that follows the stroke is the main cause of the development of spasticity, muscle contractures, flaccidity and subluxations as multifactorial causes of shoulder pain. Indeed the performance of precise routines and gradually controlled movements, lead to the decrease of these abnormal muscle tones, the disappearance of pain and the greater recovery of joint movement with the use of robotic therapy.

## Data Availability

The datasets used and analyzed during the current study are registered in the final reports from the research project “Robotic therapy for the treatment of the painful hemiplegic shoulder” at the surgical clinical Hospital “Dr. Juan Bruno Zayas” and PhD thesis of Mauricio Torres Quezada at the University of Oriente repository and are available on reasonable request.

## Abbreviations

WHO: World Health Organization
CVA: Cerebrovascular accidents
HSP: Hemiplegic shoulder pain
MAS: Modified Ashworth scale
MRC: Scale of the Medical Research Council
HSS: Humeral scapular subluxation
SHSS: Without subluxation (neurological etiology or other osteomioarticular causes)
BCI: Brain-computer interface
BMI: Brain Machine Interface
tDCs: Transcranial direct current stimulation
DOF: Degrees of freedom
ESS: Evaluation of shoulder stability
AJM: Functionality degree of active joint movement
ROM: Range of joint mobility
